# Combining metagenomics, metatranscriptomics and viromics to explore novel microbial interactions: towards a systems-level understanding of human microbiome

**DOI:** 10.1016/j.csbj.2015.06.001

**Published:** 2015-06-09

**Authors:** Shirley Bikel, Alejandra Valdez-Lara, Fernanda Cornejo-Granados, Karina Rico, Samuel Canizales-Quinteros, Xavier Soberón, Luis Del Pozo-Yauner, Adrián Ochoa-Leyva

**Affiliations:** a Unidad de Genómica de Poblaciones Aplicada la Salud, Facultad de Química, UNAM, Instituto Nacional de Medicina Genómica (INMEGEN), México, D.F. 14610, Mexico; b Instituto Nacional de Medicina Genómica (INMEGEN), México, D.F., Mexico; c Departamento de Microbiología Molecular, Instituto de Biotecnología, Universidad Nacional Autónoma de Mexico, Avenida Universidad 2001, Cuernavaca C.P. 62210, Mexico

**Keywords:** Metagenomics, Metatranscriptomics, Viromics, Human microbiome, Systems-level, Bioinformatics

## Abstract

The advances in experimental methods and the development of high performance bioinformatic tools have substantially improved our understanding of microbial communities associated with human niches. Many studies have documented that changes in microbial abundance and composition of the human microbiome is associated with human health and diseased state. The majority of research on human microbiome is typically focused in the analysis of one level of biological information, i.e., metagenomics or metatranscriptomics. In this review, we describe some of the different experimental and bioinformatic strategies applied to analyze the 16S rRNA gene profiling and shotgun sequencing data of the human microbiome. We also discuss how some of the recent insights in the combination of metagenomics, metatranscriptomics and viromics can provide more detailed description on the interactions between microorganisms and viruses in oral and gut microbiomes. Recent studies on viromics have begun to gain importance due to the potential involvement of viruses in microbial dysbiosis. In addition, metatranscriptomic combined with metagenomic analysis have shown that a substantial fraction of microbial transcripts can be differentially regulated relative to their microbial genomic abundances. Thus, understanding the molecular interactions in the microbiome using the combination of metagenomics, metatranscriptomics and viromics is one of the main challenges towards a system level understanding of human microbiome.

## 1 Introduction

The human body is inhabited by a high diversity of bacteria and archaea, as well as fungi, protozoa and viruses. These microbes inhabit in several niches within the human body sections and are collectively known as the human microbiota, whereas their collective genomes form the human metagenome [1]. The advance in high-throughput technologies used to analyze the components of the microbiome have substantially improved our knowledge of the microbial communities associated to human niches [2,3]. The decrease in cost of sequencing using high-throughput technologies has enabled large-scale studies of the human microbiome, revealing high interindividual variability of the microbiota composition and in different body sites within individuals [4–7].

Changes in abundance and composition in the fecal microbiota (dysbiosis) has been observed in patients with several human diseases, ranging from inflammatory bowel disease and obesity to diabetes and neurological disorders [8–17]. The fundamental objectives of human microbiome research is to study the structure and dynamics of microbial communities, the relationships between their members (microorganisms, viral particles and host) and their potential association with health and disease. The study of interactions between the DNAs, RNAs, and viruses that are present in the microbiome, are the main interest of metagenomics, metatranscriptomics, and viromics, respectively. For studying the microbial community of the human microbiome using high throughput sequencing technologies, there are several types of large scale analyses: the 16S profiling analysis which is based in sequencing the hypervariable regions of the 16S rRNA gene and the shotgun analysis which is based in direct sequencing of the total DNA (metagenome) and/or total RNA (metatranscriptome). In addition, the viral component of the microbiome (virome) can be also analyzed by sequencing the total viral particles.

In the last decade, many studies using the sequencing of the 16S rRNA gene to characterize the microbiota composition have been conducted, however, this analysis mainly identifies the abundance and diversity of bacteria and archaea in the sample. Although, there is a computational approach to predict the metagenome functional composition by the 16S rRNA gene sequences [18]. The metagenomic analysis also identifies the abundance and diversity of microbial community, but additionally can identify the gene content and inferred functional potential of proteins encoded in the genomes of the microbial community. The metatranscriptomic analysis allows the identification of expressed transcripts in the microbiome. The transcript numbers can also be used to compare the gene expression profiles between microbial communities. In addition, for comparative study, metatranscriptomic data must be paired with metagenomic data in order to analyze if the transcript abundance is reflecting changes in community composition [19,20]. The use of high throughput sequencing technologies to analyze human metagenomes has also revealed the existence of many bacteriophages in metagenomes [21]. Interestingly, it has been proposed that bacteriophages may have a role in shaping the diversity and composition of the oral and gut bacteria [22,23]. The involvement of phages in microbial dysbiosis may indirectly contribute to the disease. In this regard, a model suggesting that virome may contribute to the intestinal inflammation and bacterial dysbiosis was recently reported in the human gut microbiome [24]. However, studies that involve metagenomic or metatranscriptomic combined with viromic analysis are still necessary to understand the molecular interactions within the human microbiome and their relevance in health and diseased states.

The microbiome has been conceptualized as a dynamic ecological community consisting of multiple taxa each potentially interacting with each other, the host and the environment [25]. Hereafter, we use the term microbiome, to refer to the microbial communities and viruses in conjunction with the environment they inhabit, interacting as a system. In the first section of this review, an overview of the different sequencing, experimental, and bioinformatic procedures that have been used to study the human microbiome are discussed. In the second section, the recent advances combining metagenomic, metatranscriptomic and viromic analyses to identify the molecular dialog within the microbiome are dicussed. The metabolomic and metaproteomic analyses are not in the focus of this review.

## 2 Sequencing and bioinformatic strategies to study the human microbiome

### 2.1 16S rRNA gene profile analysis

**Fig. 1:**
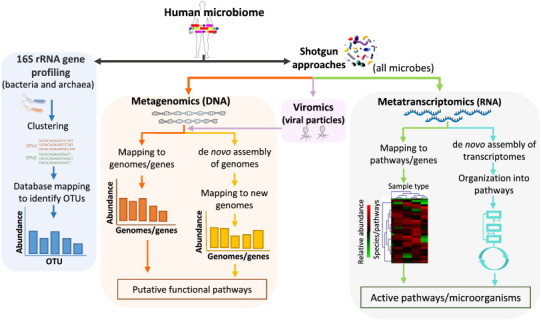
Different sequencing and bioinformatic strategies for human microbiome analysis. In the 16S rRNA gene profiling the raw sequences obtained are passed through quality filters to minimize the presence of sequencing artifacts. The resulting filtered sequence reads are clustered into operational taxonomic units (OTUs), which represent similar organisms. After that, taxonomic identity is assigned for each OTU based in sequence homology against known 16S rRNA gene databases and the relative abundance of each OTU is calculated for each sample. The resulting OTUs table is also used for quantifying population diversity within and between the samples, as the alpha and beta diversity measurements, respectively. In the shotgun approaches, metagenomic, metatranscriptomic and viromic analyses are performed. In the metagenomic analysis, the DNA sequences obtained can either be mapped to reference genomes/genes or used for *de novo* assembly of genomes. Then the relative abundance of the present genomes/genes and the functional potential of the sequences can be assessed using functional annotated databases. In viromics analysis, first the viral particles (VPs) must be enriched and posteriorly sequenced to obtain the virus genomes. Furthermore, to analyze the active genes and species of the microbiome, the metatranscriptomic analysis is applied and the obtained RNA sequences are mapped to reference pathways and genes. The results are used to identify the active pathways, genes and microorganisms. Thus, the relative abundance of each active pathway/gene/microorganism in the human microbiome is determined. The *de novo* assembly of genomes and transcriptomes can be also performed to identify novel genomes or pathways.

**Fig. 2:**
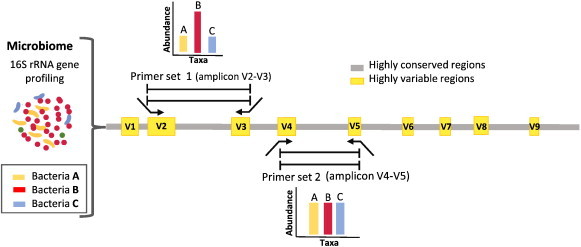
Importance of primer selection for the amplification of the hypervariable regions of the 16S rRNA gene. The figure illustrates how choosing different sets of primers for the amplification of different hypervariable regions of the 16S rRNA gene has an influence in the resulting abundance of hypothetical bacteria A, B and C. For example, in this figure, the species abundance distribution obtained using primer set 1 shows a more similar distribution to that observed in the microbiome than the abundance obtained from primer set 2. In a similar manner, Kuczynski et al [2], demonstrated that using the universal primer set F515–R806 (which is typically used to amplify a great coverage of bacteria and archea) in skin samples showed poor results for the identification of *Propionibacterium*, however the use of primer set F27–R338 was better to identify this bacteria [2].

The small ribosome subunit 16S gene (16S rRNA gene) is used as a housekeeping genetic marker to study bacterial phylogeny and taxonomy as it is highly conserved between different species of bacteria and archea. In addition to highly conserved regions, the 16S rRNA gene contains hypervariable regions that are used to identify between different bacteria. Furthermore, some bacteria have a different copy number of the 16S rRNA gene, often existing as a multigene family, or operons. Hence, the 16S rRNA gene sequencing has become typically used to identify and quantify bacterial taxa present within a microbiome sample. 16S rRNA profiling relies on using PCR ‘universal’ primers targeted at the conserved regions and designed to amplify a range of different microorganism as wide as possible. The amplified fragments (amplicons) of the gene correspond to selected short-hypervariable regions ranging from V1 to V9, making it faster and cheaper to sequence with high throughput technologies than many other bacteria genes (Figs. 1 and 2). Two of the most significant limitations of 16S rRNA sequencing that should be considered before starting a sequencing project are: (1) the introduction of biases by selection of the 16S rRNA hypervariable regions and (2) the introduction of biases by PCR primer design, which may select for or against particular groups of microorganisms (Fig. 2). To minimize the biases introduced by primer design, the primers include degenerated bases and can be used at lower hybridization temperatures to capture more microbial diversity. Other problems using the PCR is that bacterial contamination of reagents may be affecting the results [26] and that the 16S rRNA gene is also present in different copy numbers in bacterial genomes influencing the apparent relative abundance of a microorganism [26].

The sequence fragments obtained by high throughput sequencing technologies are typically called sequence reads. Longer read lengths (1000 bp), as the ones obtained by 454/Roche technology, can span multiple hypervariable regions of the 16S rRNA gene, increasing the number of the microorganisms that can be identified at species level. Although, this technology is cost prohibitive and it will be discontinued in 2016. However, short-read length sequences spanning only one hypervariable region has sufficient resolution for the accurate taxonomic assignments [27,28]. The optimal community clustering confirmed with sequence reads of this length is an important advance in amplicon design because sequencing only one hypervariable region is more cost-effective (Table 1). There is a large amount of PCR primers to amplify different hypervariable regions of 16S rRNA gene for sequencing in the short read sequencing platforms. Although, the compatibility of the fragment length should be according with sequencing platform read length capacity. There are many studies that target different regions of the 16S rRNA gene, for example V3–V5 [29], V1–V2 [30], V1–V3 [26], V4–V5 [31], and V8–V9 [32]. There is an active discussion about the hypervariable region that should be sequenced to perform a microbial diversity analysis. For example, the V6 region is not optimal for sequencing analyses that are directed for taxonomic assignment and community clustering, as opposed to sequence reads spanning the V2 and V3 regions [33,2]. The most informative 16S rRNA gene region to amplify may also depend of the analyzed environment, for example, in a study for the diagnosis of pathogenic bacteria Chakravorty at al., showed that in a mix of 110 different bacterial species including common blood borne pathogens, CDC-defined agents and environmental microflora, the V2 and V3 were most suitable for distinguishing all bacterial species to the genus level except for closely related enterobacteriaceae [34]. Additionally, the V2 region was best distinguished among *Mycobacterium* species and V3 among *Haemophilus* species [34]. Another study suggests that sequencing of the V2 and V3 regions performs well for both community clustering and taxonomic assignments in a wide range of different samples (i.e. the mouse and human gut and in the hypersaline microbial mat from Guerrero Negro). On the other hand, the Vaginal Human Microbiome Project has validated a protocol that provides species-level classification of V1–V3 16S rRNA sequences from the vaginal microbiome [35]. The primer pair 515F/R806, targeting the V4 region, was highly recommended by several human microbiome studies [20,28,36,37] and a well-established illumina protocol has been reported for this primer set [36,37]. Although no consensus has been established, the V4 region has been suggested as the gold standard for human gut microbiota characterization by MetaHit consortium [6]. Furthermore, the Earth Microbiome Project also has demonstrated that the V4 region can be extensively supported as the standard 16S rRNA region for general community assessment across a range of very different environments [38].

The most widely used bioinformatic pipelines to analyze the amplicons of the 16S rRNA gene, are QIIME [39] Mothur [40], MGRAST [41] and Galaxy [42], which are open source packages. The typical bioinformatic pipeline to analyze amplicon sequences involves three basic steps. First, the amplicon sequences are subjected to data filtering based on several quality filters. The typically used quality filters are read length, base quality (Phred score), ambiguous base calls, homopolymers, low complexity sequences and CG content. In addition, the adapter sequences should be eliminated and errors in barcodes should be corrected. Of those, it is critical to be concerned about barcode errors to avoid assigning sequence reads to the wrong sample. Second, the amplicon sequences are clustered into groups of related sequences based on their sequence similarity at a particular taxonomy level of interest (97% sequence identity is frequently chosen for species). The clusters of similar sequences are referred to as operational taxonomic units (OTUs) or sometimes phylotype, which provide a working name for groups of related bacteria (Fig. 1). OTU counts are summarized in a table of their relative abundances for each sample. Then the OTUs are compared against a reference ribosomal sequence database such as Greengenes [43], RDP [44] or SILVA [45] to assign the taxonomical classification. The third phase of analysis uses the resulting data for quantifying population diversity in the samples [46]. Within a microbial community, several measures including Shannon Index, Chao1 and Simpson's Diversity exist for calculating alpha diversity within sample. These give rise to plots of alpha diversity versus simulating sequencing effort, known as rarefraction curves. Additionally, when comparing multiple populations, beta diversity measures are applied to describe how many taxa are shared between them. In this regard, UniFrac is a beta diversity measure that uses phylogenetic information to compare the taxa shared between multiple samples and when it is coupled with standard multivariate statistical techniques including principal coordinate analysis (PCoA), identifies factors explaining differences among microbial communities [27].

### 2.2 Metagenomic analysis

Metagenomics sequencing allows the determination of the functional potential codified in the microbiome. In addition, metagenomic analyses also have been used for the discovery of novel enzymatic functions, microorganisms and genes that may be used for bioremediation [47,48], for understanding the host-pathogens interactions [49] and for novel therapeutic strategies in human diseases [50]. One challenge in metagenomic analysis is addressing the presence of host DNA in samples. For example the Human Microbiome Project (HMP) has reported high levels of human DNA in different microbiome samples, such as mid-vagina, throat and saliva samples [51,52]. The amount of host DNA varies greatly by body site and sample type, for example the samples of sputum or lung tissue in cystic fibrosis usually contain a large amount of human DNA released by neutrophils during the immune response, sometimes representing even more than 99% of the total DNA [53–55]. As a result, only a small percentage of the sequence reads from such samples correspond to microbial genomes and consequently a large percentage of sequences are eliminated. Therefore, obtaining sufficient sequence coverage of the metagenomes can become cost prohibitive. In this regard, improved experimental methods for solving the host DNA problem are needed, which are not only limited to selective cell lysis. It is also important to note that even in the absence of host DNA; the metegenomic sequencing requires a high amount of sequence to get a reasonable coverage of the microbial genomes present in the sample. Contrarily, the 16S rRNA profiling only requires a little amount of sequence to get a reasonable taxonomical census of the microorganisms present in the sample; however, it misses out the determination of gene content. A typical metagenomic experiment involves the isolation of the total DNA from the microbiome followed by its fragmentation to smaller pieces of DNA (the fragment sizes in bp depend of the selected sequencing platform). After that, the 5′ and/or 3′ ends of DNA library are repaired and adapters (containing sequences to allow hybridization to a flow cell) are ligated. The final steps are library cleanup and amplification, followed by quantification, after which the library is finally ready for sequencing.

The typical bioinformatic pipeline to analyze the sequences obtained by shotgun analysis involves as the first step the data filtering as was previously explained for 16S rRNA profiling analysis. The sequence reads that passed all quality controls are mapped to known genomes and can be also used for *de novo* assembly of contigs or genomes. However, no genomes are usually recovered for most species using metagenomic approaches. In the mapping strategy, the sequence reads are mapped (located) to a reference genome. There are powerful methods like BLAST and BLAT that are not specialized for the vast amount of data obtained by sequencing platforms. In this regard, different mapper algorithms have been developed for mapping the sequence reads to a reference genome in an efficient and productive manner, some of them are Bowtie [56], SMALT [57], BWA [58] and GEMmapper [59]. The metagenomic sequence reads are usually mapped to the human microbiome reference genome database of the Human Microbiome Project [51] and/or to specific genome databases like the Human Oral Microbiome Database (HOMD) [7] or the gut microbial gene catalog [6]. After mapping, the microbial abundance can be measured as the fraction of sequence reads that mapped to a single species in the database. Additional to the mapping strategies, there are several pipelines that compare metagenomic sequence reads against gene markers using BLAST [60], Usearch [61] or HMMs [62,63] to taxonomically annotate and quantify each metegenomic homologue. (Fig. 1). The sequence identity of the best match can be used to determine the most likely phylogenetic origin of the read. The functional diversity of the microbiome can be estimated by annotating metagenomic sequences with known functions. To this end, the sequence reads that contain protein coding genes are identified and their sequence is homology compared to the coding sequences of protein databases like the Kyoto Encyclopedia of Genes and Genomes (KEGG), protein family annotations (PFAM), gene ontologies (GO) and clusters of orthologous groups (COG). KEGG is a database resource that integrates genomic, chemical and systemic functional information [64] and PFAM is a large collection of protein families, each represented by multiple sequence alignments and hidden Markov models (HMMs) [65]. The COG database consists of clusters of orthologous groups (COG) of proteins found to be orthologous for at least three lineages and are classified into functional groups. The GO provides a controlled vocabulary of terms for describing gene product properties at three different levels: the cellular component, the molecular function and the biological process [66]. Hence, the function of the query sequence is assigned based on its similarity to sequences functionally annotated in all the above mentioned databases. The resulting data is used to describe the number of potential functions and their relative abundance in the metagenome. Furthermore, INFERNAL is a powerful tool that can be used to predict small RNA in the metagenomic data [67]. HUMAnN is an automated pipeline to determine the presence/absence and abundance of microbial pathways and gene families in a community directly from metagenomic sequence [68]. This pipeline, which is an offline platform, converts sequence reads into coverage and abundance tables summarizing the gene families and pathways in a microbial community [68]. Another offline platform used to analyze metagenomic data is the MEtaGenome Analyzer (MEGAN) [69]. Furthermore, there are integrated suites that have been designed to analyze metagenomic data sets online in an automated manner from metagenomic sequence, such as RAST (MG-RAST) [41], IMG/M server [70] and JCVI Metagenomics Reports (METAREP) [71].

In *de novo* assembly strategy, the total sequence reads are used to assembly genomes (Fig. 1). Additionally, the assembly can be also performed only using the sequence reads that were not mapped to known genomes (Fig. 1). There are several bioinformatics tools used for *de novo* assembly like MetaVelvet [72], khmer [73], metamos [74], Meta-IDBA [75], MetaORFA [76] and RayMeta [77]. Metagenomic assemblers generally adapt graph-based reconstruction approaches as the overlap–layout–consensus (OLC) to assemble longer sequences and de Brujin graph to assemble shorter sequences. However, the assemblers based in the de Brujin graphs are the most used due to the success of the shorter sequences produced by popular sequencing platforms of Illumina and Life Technologies companies (Table 1). After that, the new assembled genomes can be used for mapping the metagenomics sequence reads to estimate the abundance of these new genomes in the microbiome. Furthermore, the new genomes are also used for functional annotation when compared against sequences annotated in databases as KEGG or GO (Fig. 1).

### 2.3 Metatranscriptomic analysis

Metagenomics is a powerful tool used to describe the gene content and potential functions encoded in sequenced genomes. However, metagenomics approaches have a very limited role in revealing the microbial activity measured by gene expression. The metatranscriptomic shotgun sequencing (RNAseq) provides the access to the metatranscriptome of the microbiome allowing the whole-genome analysis profiling of the active microbial community under different conditions. In this regard, sequencing of metatranscriptomes has been recently employed to identify RNA-based regulation and expressed biological signatures in human microbiome [78]. However, only few investigations (which are discussed in the second section of this review) have performed a combined analysis using metatranscriptomics with metagenomics. A typical metatranscriptomic experiment involves the isolation of the total RNA from the microbiome followed by RNA enrichment depending on the type of RNA to be sequenced (i.e. mRNA, lincRNA, and microRNA). After that, the RNA is fragmented to smaller pieces (the fragment sizes in bp depend of the selected sequencing platform) followed by cDNA synthesis using reverse transcriptase and random hexamers or oligo(dT) primers. After that, like in the construction of metagenomic libraries, the 5′ and/or 3′ ends of the cDNA are repaired and adapters are ligated, followed by library cleanup, amplification and quantification and finally the library is sequenced. As converting RNA into cDNA has been shown to introduce biases in quantification of transcripts [79], semi direct RNA sequencing without cDNA synthesis has been developed [80–82]. There are several technical issues affecting the large-scale application of metatranscriptomics: (1) the collection and storage procedures to preserve the RNA of the sample, (2) the limitation to obtain high-quality and sufficient quantity of RNA from human microbiome samples, (3) the mRNA enrichment procedures by removing ribosomal RNA (rRNAs) which represent over 90% of the RNA, (4) the average useful life of mRNA leads to difficulty in the detection of rapid and short-term responses to environmental changes, (5) the transcriptome databases are insufficient, (6) the host RNA contamination which cannot be removed by currently available rRNA purification methods and (7) the poly-A RNA selection kits to capture the mRNA population are not feasible in prokaryotes. However, several efforts have been recently made to tackle these technical issues, for example Franzosa et al. have developed experimental strategies to improve the point 1 [19] and Giannokus et al. [83] have analyzed strategies to improve points 2 and 3. Interestingly, the Ambion's MICROBEnrich Kit uses hybridization capture technology to remove human, mouse, and rat RNA (both mRNA and rRNA) from complex host-bacterial RNA populations, leaving behind enriched microbial total RNA.

The typical bioinformatics pipeline to analyze the data obtained from a metatranscriptomic experiment is similar to the one used in metagenomics and it is also divided in two strategies: (1) mapping sequence reads to reference genomes and genes and (2) *de novo* assembly of new transcriptomes (Fig. 1). In the first strategy, after mapping the RNA sequence reads to different genomes or pathways is possible to identify the taxonomical classification of active microorganism and the functionality of their expressed genes. For example, through the mapping of metatranscriptomic sequences to KEGG database [64], the pathways whose expressed genes are up and down regulated or unchanged in the microbiome during health and disease conditions are obtained [84]. There are several bioinformatic programs used in metagenomics like SOAPdenovo [85], ABySS [86] and Velvet-Oases [87] that have been reported to be successfully applied to the metatranscriptome assembly of microbiomes [86,88–91]. However, Trinity is a program specially developed for *de novo* transcriptome assembly from short-read RNA-seq data and it is a very efficient and sensitive in recovering full-length transcripts and isoforms and now is one of the most used bioinformatics tools to assembly *de novo* transcriptomes of very different species [92–94].

### 2.4 Viromic analysis

Viruses outnumber microbial cells 10:1 in most environments; however, viral DNA only represents 0.1% of the total DNA in a microbial community [6]. Hence, to obtain a deep sequence coverage of the human viruses that are present in the microbiome, the isolation of viral particles (VPs) becomes necessary [95–98]. In solid samples containing viruses, such as human feces, a common approach is to suspend the fecal material in an osmotic neutral buffer followed by serial filtration steps to remove large particles, including undigested or partially digested food fragments and microbial cells [99]. Additionally, other protocols use the ultracentrifugation with a cesium chloride density gradient to separate the viral particles from the microbial cells [100,101]. After that, viral particles are purified and the non-encapsulated free nucleic acids are removed by treatment with DNase and RNase, then the VP-derived nucleic acids are isolated. However, because the amount of DNA extracted from purified VPs is often below the required for sequencing, the amplification of the total viral DNA is typically necessary. To this end, a range of amplification methods have been developed, such as random amplified shotgun library (RASL) [102], linker-amplified shotgun library (LASL) [103] and the Multiple Displacement Amplification (MDA) [104]. The MDA method takes the advantage of the high processivity of the phage-derived φ29 polymerase which synthetize > 70,000 nucleotides per association–dissociation cycle and its strong strand displacement capability, which together allow the amplification of complete viral genomes. Although, recent publications have shown that critical biases and contamination are introduced to the sample when the MDA amplification is used [105–109].

The sequencing technologies that prioritize long read lengths over those of short read lengths are preferred, because most viral sequences are novel and they are enriched in regions of low-complexity repeats [110,111]. However, the sequencing technologies of long read lengths as 454/Roche pyrosequencing are about to be discontinued. To resolve this limitation many bionfomatic programs have been developed to analyze viruses from short sequence reads [27]. The initial analysis of the sequences obtained after DNA sequencing of VPs also involves the data quality filtering like the one in the 16S rRNA profiling and metegenomic sections. However, the majority (usually, 60–99%) of sequences in viromes from any environment have no significant similarity to other sequences in databases or have higher homology to prokaryotic or eukaryotic genes [112,103,113–116], therefore, the filtering of bad quality sequences and the decontamination of 16S rRNA, 18S rRNA and human sequences by mapping is still important in viromic analysis. The resulting sequences are compared against individual viral genomes using several mapping algorithms or using programs as BLASTX or USEARCH (as were previously described in the metagenomic section), to analyze the taxonomic composition of viral community.

Although viral sequence databases have considerably expanded due to the start of the viromics era, the number of deposited genomes is far less than the expected number of virotypes [117] and most of the new sequences are poorly annotated [118]. Furthermore, the percentage of sequence reads with similarity to known viral sequences depends on how well the sequences have been filtered and the database that is used, however, it is generally less than 0.01% [119–121]. Although, the number of sequence reads with similarity against databases of viral genomes depends on how well the sequences have been filtered and the database that is used for comparison. There are several databases focused on taxonomical virus classification, such as the Classification of Mobile Genetic Elements (ACLAME) [122] and Phage SEED [123]. However, is important to note that ACLAME database has not been updated in the last years. After taxonomic and functional assignments a viral community profile characterizing the diversity in the sample is created. However, given that most of the available viral metagenomic data lacks similarity to sequences in the databases, similarity-independent methods have been developed to better understand viral community structure. One example of that is Phage Communities from Contig Spectrum (PHACS), which is a bioinformatic tool to assess the biodiversity of uncultured viral communities. PHACS was designed to quantify virotypes [112,124] based on the assumption that if a virotype is present in high abundance in a VP sample it is more likely to be assembled into a large contig. Another alternative for identifying shared viruses among different samples is crass [125], an algorithm that allows the simultaneous cross-assembly of all the samples in a data set as opposed to the pairwise assemblies used in MaxiPhi [114], which is based on contig assemblies generated from the pooled VP viromes. The chimeras are a common problem with most assemblers, although most occur between viruses and not so much between virus and bacteria. In this regard, the overlap–layout–consensus (OLC) algorithms have showed efficiency in the viral genomes assembly. One of the most popular of these assemblers is Newbler which has been extensively used in viral and bacterial shotgun metagenomic projects [6,95,126–131]. However, it remains to be determined if Newbler will be discontinued with the 454/Roche in 2016. Additionally, two other new OLC assemblers were developed and tested on viral metagenomics data: Minimo, designed for the assembly of small datasets [132] and used for virome analyzes [95,133]; and VICUNA, an assembler specialized in *de novo* assembly of data from heterogeneous viral populations [134]. De Brujin graph assemblers, as MetaVelvet [72], are an alternative strategy to the OLC assemblers and have also been used on the assembly of viral metagenomes [135]. Other popular metagenome assemblers for viromes are IBDA [136] and RayMeta [77]. The RNA viruses in the human gut microbiome are mostly influenced by RNA from ingested plants of the food [89], therefore, the sequencing of total RNA viruses is impractical. However, it remains to be seen whether they represent stable members of the gut virome or are transiently present as a result of plant consumption [89]. Finally, in the Table 1 are summarized the most common high throughput sequencing technologies used in 16S rRNA profiling and shotgun approaches.

## 3 Characteristics of oral and gut microbiomes

The oral and gut microbiomes represent the two best-studied human microbiomes to date. The human gastrointestinal tract involves an extremely complex and dynamic microbial community, that includes archaea, bacteria, viruses and eukaryote [3]. However, most of the microorganisms that inhabit the gastrointestinal tract are bacteria and 70% of them inhabit the colon [137]. The gut microbial community plays an important role in protecting the host against pathogenic microbes [138,139], modulating immunity [140,141] and regulating metabolic processes [142,143]. Also, the human gut has been considered as a neglected endocrine organ [144]. In the gut microbiome, bacteria can interact with each other while they must also compete with each other and phages are expected to have a significant role in driving the biodiversity of this complex ecosystem. Even if the role of human gut microbiome has been well reviewed at a metagenomic level [145–147], its integration with viromic and metatranscriptomic analyses has been little studied yet.

The human oral cavity is the second most important niche of the human body, due to the enormous amount of microorganisms that inhabit it like viruses, fungi, protozoa, archaea and bacteria. The most representative microorganisms at the oral cavity are the bacteria and viruses and their different abundances and composition have been associated with several dental diseases [148]. The bacterial diversity in the oral microbiome is approximately of 1000 species where the most representative phyla are Firmicutes, Bacteriodetes, Proteobacteria, Actinobacteria, Spirochaetes and Fusobacteria.

### 3.1 Metagenomic and 16S rRNA profiling combined with viromic analyses

Over the past decade, an increasing number of studies have indicated that changes in bacterial abundance and/or composition are associated with the presence of several human diseases [1,16,137]. Bacteria are the most enriched microbes inhabiting human body sites and their viruses (bacteriophages) are significantly more prevalent than eukaryotic viruses [103,149]. The bacteriophages (phages) are a natural antibacterial able to regulate bacterial populations due to the induction of bacterial lysis or by providing functional advantages to their host [150] (Fig. 4a). According to this, the application of phages to the treatment of chronic bacterial infections has proved their potential role in human health by taking advantage of their capacity to destroy pathogens [150,151]. However, there are very few examples studying the interaction between phages and their microbial hosts in human microbiome combining viromics and metagenomics. In this regard, Norman et al. used 16S rRNA gene profiling and virome sequencing to suggest that decreased diversity of enteric bacterial community observed in patients with Crohn's disease and ulcerative colitis was associated with an abnormal virome composition [24]. In particular, the bacteria family *Bacteroidaceae* showed a reduction in their relative abundance that was correlated with an increase in several *Caudovirales* bacteriophages in Crohn's disease (CD) [24]. In contrast, the families *Enterobacteriaceae* and *Pasteurellaceae* were increased in their relative abundance in patients with CD [24]. Although other studies based only on 16S rRNA profiling or metagenomic analyses [152–154] also showed a low bacterial diversity in inflammatory bowel disease, the Norman et al. study demonstrated that bacteriophages may have a role in this disease through interactions with the bacterial community of the microbiome [24]. This study shows how powerful it is to combine the viromics with 16S rRNA profiling for a better comprehension of the human microbiome.

**Fig. 3:**
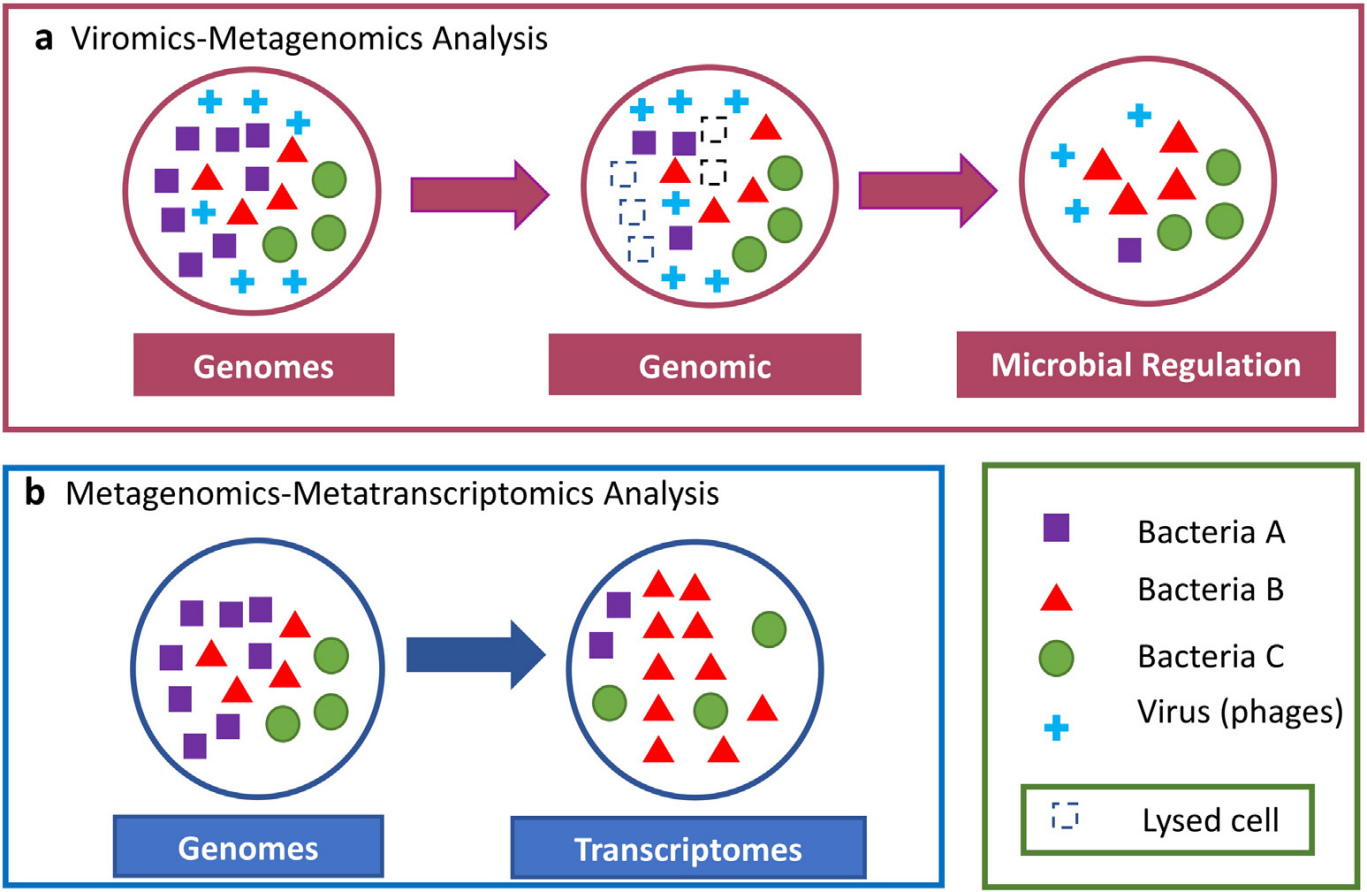
Molecular interactions explored using metagenomics–viromics and metagenomics–metatranscriptomics analyses. The interactions between microorganisms in the human microbiome can be better studied combining omics analysis. (a) In this panel is illustrated how phages can interact and affect the microbial diversity by infecting their host bacteria and thus promoting homeostasis or disbyosis [179,24]. This type of interaction can be explored using viromics combined with metagenomics. (b) The species abundance of the three hypothetical bacteria can be different depending on the used analysis (metagenomics or metagenomics combined with metatranscriptomics) [171]. The data integration and normalization when metagenomics is combined with metatranscriptomics is important because the metatranscriptomics data can correspond to different species abundance and/or to differentially expressed transcriptomes.

Other studies in the gut and oral microbiomes through the integration of metagenomics and viromics have shown that virus diversity correlates with their microbial host counterparts [21,95,100,127,155–158]. For example, the analysis of all publicly available fecal metagenomes showed that the new bacteriophage crAssphage was the predominant phage in all samples [21]. Dutilh et al. predicted that the crAssphage host belongs to Bacteroidetes phylum and they found that this virus comprises up to 90% and 22% of all sequence reads in viral particle derived metagenomes and total community metagenomes, respectively [21]. Interestingly, the 90% of the human gut microbiome comprises some combination of Bacteroidetes and Firmicutes, hence Dutilh et al. suggested, that a high amount of crAssphage can be due to the high proportion of their host in the human gut microbiome [21]. Another study that also showed a strong correlation between the bacterial diversity (measured by 16 s rRNA sequencing) and viral diversity (measured by VPs sequencing) in gut microbiome is the one published by Minot et al. [100]. The study showed that correlation on diversity is maintained over time (1–8 days), suggesting that the observed stability in bacterial diversity is also reflected in their host viruses through time [100]. Interestingly, the functional potential reflected by the 16S rRNA gene profiling was different to the one observed by VPs sequencing of the same sample. However, whether the changes in phage abundance are a result of changes in abundance of their hosts, or whether additional mechanisms (as lysogenic induction) are involved will require further work combining metagenomics with viromics data (Fig. 3a).

Interestingly, it was demonstrated that phages can accelerate the genomic evolution of its bacterial host in the microbiome [159,160] and these genomic changes can lead to functional adaptations of host's bacterial community [156]. In this regard, it has been demonstrated that gut and oral viromes are dominated by temperate phages integrated into the genome of their hosts, therefore this integration may alter host-bacterial phenotype by lysogenic conversion [95,100,161,162]. An example of that functional interaction was recently demonstrated in an animal model, where a prophage was liberated from its host cell after that cell was exposed to a fecal community [95]. The authors determined that after two weeks of colonization of gnotobiotic mice with two bacteria containing prophages those were differentially activated in cecal and fecal samples [95]. Moreover, a differential prophage induction was also observed in human fecal samples [100] by comparing metagenomic with viromic sequences [100]. The later study also suggests that viruses are a reservoir for functions that can be used by their host under specific biological conditions, like stress or diet (Fig. 3a). There are multiple ways in which a prophage offers evolutionary advantages to their hosts through genetic diversity [163]. In this regard, a recent study demonstrated that activation of the composite phage (0 V1/7) in *E. faecalis* is controlled by the nutrient availability in the mouse intestine [156]. This study suggests that prophages can impact the dynamics of bacterial abundance in the mammalian gut after the exposition to nutrient availability [156]. Furthermore, the phage-encoded proteins can increase virulence either indirectly by aiding bacterial adaptation to the niche and/or directly through expression of toxins and other virulence factors [164–166]. For example, in oral microbiome the virulence of *Corynebacterium diphtheria* was associated with a small region of a putative prophage [167].

The articles discussed above demonstrated that microbial abundance and composition in the gut and oral microbiomes are also influenced by phage members and that virus activation can be triggered by the human diet and habits. This suggests that virus–bacteria interaction could be present in complex diseases, like obesity and diabetes. However, the genetic regulation that bacteria offer against the viruses is also important. For example, the CRISPRs (Clustered Regularly Interspaced Short Palindromic Repeats) which are spacer sequences that interfere with viral replication have been detected in human microbiome [168,169]. This suggests that CRISPRs mechanism contribute to the interaction between bacteria and viruses in the human microbiome. Hence, the interactions between phages and their hosts are of great interest for understanding their impact in shaping the abundance and composition of the human microbiome [158]. However, further studies are required to understand if the interaction between metagenomes and viromes play a significant role in the progression or impediment of human diseased states associated with changes in microbiome abundance and composition.

The most common microorganism used as a probiotic is Bifidobacteria. However, in the majority of Bifidobaterial genomes the existence of prophage sequences has been confirmed, suggesting that also these intestinal commensals are targeted by phage predation [170]. Interestingly, in probiotics trials there are always subsets of individuals who do not respond to probiotics interventions, leading us to think about the role that the interaction of phages–bacteria and/or the inter-individual differences in bacterial composition are playing in shaping the microbial populations under probiotics treatments. However, more detailed investigation on the interactions between phage, bacteria and probiotics are necessary [170]. For example, determining the virome combined with metagonome before and after probiotic treatment may be an effective method to study the dynamics of gut microbial community.

### 3.2 Metagenomic combined with metatranscriptomic analyses

**Fig. 4:**
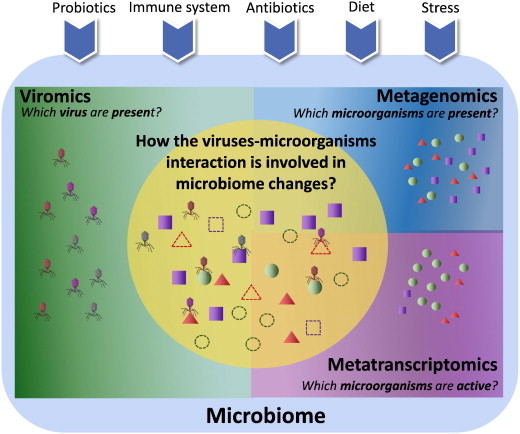
Towards a systems level understanding of human microbiome. The use of only one analysis to study the human microbiome (viromics, metagenomics or metatranscriptomics) provides a partial view of the complete ecological system. In a combined approach, the metagenomic analysis can give us a view of the microorganism's abundance and functions available in the microbiome, while the metatranscriptomic analyses combined with metagenomics can show us which of these microorganisms and functions are actually active. Finally, the integration of viromics analysis with the other omics data can provide information about the role that viruses play within the microbiome. The combined analyses can offer a better understanding of the role that external factors like diet, immune system and probiotics are playing in shaping the human microbiome abundance and composition. Thus, an integrated systems analysis (orange circle) seems necessary to have a better understanding of molecular mechanisms and their interactions in human microbiome.

The metatranscriptomics is becoming increasingly practical as a tool to analyze the regulation and dynamics of transcriptionally active microbial community. The application of metatranscriptomic combined with metagenomic analysis has showed that gut microbiome contains distinctive sets of active microorganisms between individuals [19,171] (Fig. 4b). Interestingly, the induction of microbial genes as a response to host targeted exposure of xenobiotics has been observed using metagenomic combined with metatranscriptomic analysis of the gut microbiome [172]. The later study also shows that the level of metabolic activity can define the gut microbiote members [172]. However more studies are necessary to understand how metabolic activity can influence bacterial fitness and how external factors like diet are implicated in their expression (Figs. 3a and 4). The potential of combining metatranscriptomic with metagenomic analysis is clearly observed in the Franzosa study [19]. This study showed that a substantial fraction (59%) of microbial transcripts was differentially regulated relative to their genomic abundances. The study also demonstrated that several gene families that are less abundant at the metagenomic level can be very active at the metatranscriptomic level and vice versa [19], suggesting that performing metagenomic analyses alone, could overestimate or underestimate the functional relevance of the encoded genes in metagenomes [19]. The authors also suggest that functional diversity at transcriptional level shows a pattern of subject-specific metagenome regulation; this is measured by the function of the top 10 gene families of each analyzed individual [19]. Interestingly, the metatranscriptomics profiles were significantly more individualized than DNA-level functional profiles, suggesting a subject-specific whole-community regulation. Additionally, the Franzosa study also demonstrates that the gut microbiome seems to be more stable between samples at a functional level independently of the microorganisms that are producing them. Another recent study performed by Gosalbes et al. [173], sequenced the total RNA of 10 fecal samples from healthy volunteers and the relative abundance at family level of the 16S rRNA sequences indicated that phylogenetic composition is not uniformly distributed among individuals. Contrarily, the functional analysis of putative mRNAs against COG database showed and increased homogeneity distribution of the functional COG categories between the samples [173]. Interestingly, Gosalbes et al. suggest a health related functional profile showing some differences with those indicated by the potential functions of predicted genes in DNA-based surveys. In another work on oral microbiome, the relative abundance of bacterial genera with metagenomic data was also different to that obtained with metatranscriptomic data [171].

The studies discussed in this section suggest that metatranscripomic combined with metagenomic analyses allow a deep understanding on microbial interactions within human microbiome. Furthermore, these combined omics analyses provide useful insights about the microorganisms that have relevant functions and at the same time it allows knowing the active genes and pathways that can be related to a diseased phenotype (Fig. 4). Furthermore, the interactions between prophages and bacteria within the human microbiome can also be explored through the identification of the expressed genes from the prophages under particular ambient conditions (Fig. 4). Applying such approach to study the expression of phage genes in the gut microbiome can indicate the role of encoded prophage genes in microbial physiology and determine the dynamics that exist between active phages and their microbial host.

## 4 Perspectives and future trends of the human microbiome analyses

The study of human microbiome through the combination of metagenomic, metatranscriptomic and viromic analyses allows a deep understanding of molecular interactions within microorganisms and their role in human health and disease (Fig. 4). Of these combined analyses the identification of potential genes, pathways and viruses that can be associated with health and disease is also possible. These driver genes and pathways could be explored as a potential pharmacological target to treat diseases that are associated with a microbiome dysbiosis. Although bacteria have been directly associated with human diseases, the role of the virome in the microbial community should be explored. However, the lack of a conserved region in virus genomes (like the 16S rRNA gene of bacteria), make the study of viruses more difficult to analyze in large cohorts. Hence, the development of novel experimental and bioinformatic strategies for a better comprehension of the role that viruses play in the microbial dysbiosis is necessary.

Probiotics can profoundly alter the human microbial community [170–175], opening the possibility to use them for the treatment of diseases associated with microbial dysbiosis. However, the reestablishment of microbial diversity has not been observed in all individuals subjected to probiotic therapy trials [170]. [176,177]. Therefore, the combination of metagenomics, metatranscriptomics and viromics is an important approach to investigate the role that the interaction between viruses and bacteria play in probiotic therapies. The systems level study of human microbiome opens the opportunity to identify novel molecular targets for the treatment of microbial dysbiosis associated to human diseases.

## Figures and Tables

**Table 1:** Typical high throughput sequencing platforms used in 16S rRNA gene profiling and shotgun sequencing approaches.

Sequence read length	Hypervariable regions that can be evaluated	Shotgun utility	Costs	Sequence reads per run	Run time
Platform: Roche 454 GS-FLX +					
800 base reads	Up to seven per read; long reads allow a good coverage of 16S rRNA gene, allowing a good taxonomical assignment.	Long reads help with assembly of new genomes.	Cost limits the deep sequencing analysis.	1 million	20 h
Platform: Illumina HiSeq, MiSeq and NextSeq					
100–500 base reads	Only one per read (NextSeq) Up to 3 per read (HiSeq and MiSeq); short reads do not seem to limit the taxonomical analysis.	Short reads, but high sequencing output allows a deep sequencing analysis.	Cost-effective deep sampling	25 million, 2 × 300 bp (MiSeq) 130–400 million, 2 × 150 bp (NextSeq) 2.5 billion, 2 × 150 bp (HiSeq)	5–55 h (MiSeq) 12–30 h (NextSeq) 24–84 h (HiSeq)

Platform: Life Technologies ion personal genome machine (PGM)					
35–400 base reads	Up to 3 in custom design. Up to seven using the Ion 16S metagenomics kit; in a very fast sequencing time.	Short reads, but mid sequencing output in a very fast sequencing time.	Time-effective with a mid-cost for deep sequencing analysis.	1 × 200 or 1 × 400 and number of reads depends on model of the sequencing chip: 10 million (314) 100 million (316) 1 billion (318)	2–4 h (314) 3–5 h (316) 4–7 h (318)
